# Utility of Prophylactic Percutaneous Gastrostomy in Patients With Head and Neck Cancer Receiving Concurrent Chemoradiotherapy: A Multicenter Analysis

**DOI:** 10.7759/cureus.44637

**Published:** 2023-09-04

**Authors:** Maria Teresa Neves, André Ferreira, Vanessa Branco, Maria Abreu, Fátima R Alves, Carlota Baptista, Joana Graça, Filipa Ferreira, Mariana Malheiro, Ana Martins

**Affiliations:** 1 Medical Oncology, Hospital São Francisco Xavier, Centro Hospitalar de Lisboa Ocidental (CHLO), Lisbon, PRT; 2 Medical Oncology, Hospital Beatriz Ângelo, Loures, PRT; 3 Medical Oncology, Hospital de Vila Franca de Xira, Vila Franca de Xira, PRT; 4 Medical Oncology, Hospital CUF Tejo, Lisbon, PRT

**Keywords:** prophylactic, prophylactic percutaneous gastrostomy, nutrition, head and neck cancer, locoregionally advanced, chemoradiotherapy

## Abstract

Introduction: Patients with head and neck cancer (HNC) have an elevated incidence of cachexia and malnutrition due to the tumor's location interfering with oral feeding. Concurrent chemoradiation (CCRT) can have an emetic effect and cause dysphagia and oral mucositis. Adequate nutrition improves immunity, raises the response to therapy, reduces adverse effects, and improves survival. Numerous studies have suggested the utility of nutritional support from percutaneous endoscopic gastrostomy (PEG) in HNC patients. Although PEG is usually considered a safe procedure, it has a mortality rate of 0-2.2% and a risk of other procedure-related complications of 17-40%. Our work intends to evaluate the utility of PEG in patients with locally advanced HNC who underwent CCRT.

Methods: We performed a cohort study at three institutions. We included patients with HNC who underwent definitive CCRT treatment from January 2013 to December 2022. The study consisted of an observational, descriptive, retrospective analysis of prespecified clinical data. Descriptive statistics were used to compare the data between the PEG group and the non-PEG group. Analysis of covariance (ANCOVA) was used for covariance analysis. Fisher’s exact test was used to compare proportional data and Student's t-test was used to assess the differences in continuous data. Survival analysis was performed using the Kaplan-Meier estimator. P-values of <0.05 were considered to be indicative of statistical significance. The SPSS Statistics version 28.0 (Armonk, NY: IBM Corp.) was used to perform all statistical evaluations.

Results: We identified 90 eligible patients diagnosed with local advanced HNC who had received definitive CCRT with three weekly cycles of cisplatin as follows: 44 with a prophylactic PEG tube and 46 without a prophylactic PEG tube. Most patients were male (84.4%) and 50% of patients were diagnosed with stage IVa HNC at the time of diagnosis. There wasn't an effect of PEG placement on BMI at the end of CCRT after controlling for the effect of baseline BMI (F {1.84}=0.065 {p=0.799}). In the study population, BMI was significantly lower after CCRT (21.30 kg/m^2 ^vs. 23.97 kg/m^2^), t (86)=12.389, p<0.001. In the subgroup with baseline BMI <18.5 kg/m^2^ (15 patients), 90% of patients with prophylactic PEG were able to complete the three planned cycles of chemotherapy vs. 66.7% in the non-PEG group. Ten patients in the PEG group (22.7%) referred feeding tube dependency. Patients with dysphagia were 3.2 times more likely to have placed prophylactic PEG (p=0.007). The difference in overall survival and progression-free survival between the two groups was not statistically significant (p=0.57 and p=0.497, respectively).

Conclusion: In this study using real‐world data, we found a potentially protective effect of PEG in underweight patients with locally advanced HNC performing CCRT in order to complete three cycles of treatment.

## Introduction

Head and neck cancer (HNC) is the sixth most frequent cancer type worldwide, with over 870,000 new cases and 440,000 deaths in 2020 [1]. Patients with HNC have an elevated incidence of malnutrition due to the tumor's location interfering with oral feeding and due to treatment toxicity [2]. Cancer cells secrete cytokines, such as tumor necrosis factor-α, interleukin-1, and interleukin-6, which often lead to energy consumption, metabolic abnormalities, and loss of skeletal mass which can cause anorexia and cachexia in these patients [3,4]. Furthermore, concurrent chemoradiotherapy (CCRT) can have an emetic effect and oral mucositis, hence, causing dysphagia, which all lead to weight loss. Patients with HNC who are afflicted by malnutrition experience a two-year survival rate of 7.5% compared to 57.5% for those without malnutrition [5]. Therefore, ensuring proper nutrition during HNC treatment becomes a crucial objective. This approach not only enhances treatment response and survival but also bolsters immunity and mitigates adverse effects [6].

National Comprehensive Cancer Network (NCCN) guidelines recommend the placement of a prophylactic percutaneous endoscopic gastrostomy (PEG) for HNC patients with considerable weight loss prior to treatment (5% weight loss over the previous one month or 10% weight loss over six months); risk of aspiration; dysphagia; anorexia or pain interfering with the ability to receive adequate oral intake, especially if the patient presents comorbidities that may be aggravated by poor tolerance to dehydration; lack of caloric intake; or difficulty in swallowing necessary medications [7]. PEG is a widely used procedure that is minimally invasive. In order to enhance treatment tolerance, prevent weight loss, and improve survival in patients with HNC, prophylactic PEG tube insertion is increasingly performed before CCRT [8]. Numerous studies have proposed the utility of this nutritional support [8-17].

Although the placement of PEG is usually considered a safe procedure, it may have complications, such as local wound infection or inflammation, abdominal pain, and bleeding in 17-29% of cases and has a mortality rate of 0-2.2% [18]. It can even lead to feeding tube dependency, secondary to swallowing dysfunction [19-22].

At our institutions, and according to NCCN clinical practice guidelines, before starting CCRT patients with HNC have the option to place prophylactic PEG. Patients are informed about the risks and benefits of this procedure and the final decision is based on their preference. Some patients consider the placement of prophylactic PEG useless because they are still able to feed themselves orally during CCRT [18]. For this reason, we intended to evaluate the use of prophylactic PEG in patients with locally advanced HNC receiving CCRT with real-world data and identify a potential subgroup of patients that benefit the most from this procedure.

This article was previously presented as an abstract at the MASCC/JASCC/ISOO Annual Meeting 2023 on June 22, 2023, and subsequently published in the journal, Supportive Care in Cancer, volume 31, supplement issue 1, June 2023 (https://doi.org/10.1007/s00520-023-07786-4).

## Materials and methods

We performed a cohort study across three institutions. We included patients with HNC who underwent definitive CCRT treatment from January 2013 to December 2022. The study was conducted in accordance with the Declaration of Helsinki principles of good clinical practice. All patients provided written, informed consent before inclusion.

The study consisted of an observational, descriptive, retrospective analysis of prespecified clinical data. This data was collected from individual electronic medical records, including medical and nursing records. The study's inclusion criteria were patients aged 18-100 years; diagnosis of stage III-IVb HNC, according to the eighth edition of the American Joint Committee on Cancer Staging System; submitted to definitive CCRT at the nasopharynx, oral cavity, oropharynx, hypopharynx, or larynx; treatment with three weekly cycles of cisplatin chemoradiotherapy; and no previous cancer treatment regimen. A radical dose of radiation (66-70 Gy) was planned for all patients. The exclusion criteria were patients with distant metastasis or previous radiation and/or chemotherapy. We collected and analyzed the patients' clinical characteristics, including age; sex; tumor site; tumour, node, and metastasis (TNM) staging; histopathology; treatment regimen; Eastern Cooperative Oncology Group score; presence of dysphagia symptom; body mass index (BMI); nutritional status; assessment in nutrition appointment; presence or absence of PEG tube; hospitalization during CCRT; number of cycles of chemotherapy; PEG tube use; duration of PEG tube use; progression of disease; and death. After discussing each case in a multidisciplinary tumor board, treatment strategies were based on institutional protocols following the European Society for Medical Oncology (ESMO) and NCCN guidelines. Prophylactic PEG tube placement was generally considered for all patients with HNC undergoing CCRT. Nevertheless, the overall risks and benefits of this procedure were discussed with patients, and the final decision was based on the patient's preference. Patients were divided into two groups: the PEG group and the non-PEG group.

All people involved in data collection were trained to standardize this procedure. Descriptive statistics were used to compare the data between the two groups of patients. Analysis of covariance (ANCOVA) was used for covariance analysis. Fisher’s exact test was used to compare proportional data and Student's t-test was used to assess the differences in continuous data. Survival analysis was performed using the Kaplan-Meier estimator. We considered having statistical significance p-values of <0.05. The SPSS Statistics version 28.0 (Armonk, NY: IBM Corp.) was used to perform all statistical evaluations.

## Results

We identified 90 eligible patients diagnosed with locally advanced HNC who had received definitive CCRT with three weekly cycles of cisplatin as follows: 44 with a prophylactic PEG tube and 46 without a prophylactic PEG tube. The patient demographics and their baseline disease characteristics are shown in Table 1.

**Table 1:** Baseline clinical characteristics. ECOG: Eastern Cooperative Oncology Group; CCRT: concurrent chemoradiation; SCCA: squamous cell carcinoma

Clinical characteristics		Non-PEG group (n=46)	PEG group (n=44)	All (n=90)
Age (year) mean (SD)		58.83 (9.689)	60.32 (8.852)	59.56 (9.26)
Sex	Female, n (%)	7 (15.2)	7 (15.9)	14 (15.6)
	Male, n (%)	39 (84.8)	37 (84.1)	76 (84.4)
ECOG PS, n (%)	0	37 (80.4)	30 (68.2)	67 (74.4)
	1	9 (19.6)	14 (31.8)	23 (25.6)
Histopathology, n (%)	Non-keratinizing SCCA	3 (6.5)	0 (0.0)	3 (3.3)
	Keratinizing SCCA	43 (93.5)	44 (100.0)	87 (96.7)
Tumor sites, n (%)	Oral cavity	4 (8.7)	4 (9.1)	8 (8.9)
	Oropharynx	19 (41.3)	23 (52.3)	42 (46.7)
	Hypopharynx	3 (6.5)	5 (11.4)	8 (8.9)
	Larynx	14 (30.4)	10 (22.7)	24 (26.7)
	Nasopharynx	6 (13.0)	2 (4.5)	8 (8.9)
T classification, n (%)	1	2 (4.3)	2 (4.5)	4 (4.4)
	2	11 (23.9)	7 (15.9)	18 (20.0)
	3	23 (50.0)	15 (34.1)	38 (42.2)
	4	10 (21.7)	20 (45.5)	30 (33.3)
N classification, n (%)	0	20 (43.5)	11 (25.0)	31 (34.4)
	1	8 (17.4)	7 (15.9)	15 (16.7)
	2	14 (30.4)	20 (45.5)	34 (37.8)
	3	4 (8.7)	6(13.6)	10 (11.1)
Clinical staging, n (%)	III	23 (50)	14 (31.8)	37 (41.1)
	IVa	20 (43.5)	25 (56.8)	45 (50)
	IVb	3 (6.5)	5 (11.4)	8 (8.9)
Symptom of dysphagia, n (%)	No	29 (63.0)	15 (34.1)	44 (48.9)
	Yes	17 (37.0)	29 (65.9)	46 (51.1)
	Baseline BMI, kg/m^2^ mean (SD)	24.70 (5.76)	23.20 (5.34)	23.97 (5.58)
Nutritional status (based on BMI), n (%)	<18.5	5 (10.9)	10 (22.7)	15 (16.7)
	18.5-24.9	21 (45.7)	16 (36.4)	37 (41.1)
	25-29.9	12 (26.1)	13 (29.5)	25 (27.8)
	≥30	8 (17.4)	5 (11.4)	13 (14.4)
Assessment in nutrition consultation, n (%)	No	12 (26.1)	7 (15.9)	19 (21.1)
	Yes	34 (73.9)	37 (84.1)	71 (78.9)

Most patients were male (84.4%), with a mean age at diagnosis of 56.56 (±9.26) years. Fifty percent of patients in this cohort were diagnosed as stage IVa at the time of diagnosis. There is a significantly higher proportion of patients with dysphagia in the PEG group (Fisher's test, p=0.007). The majority of patients received three cycles of chemotherapy with cisplatin (70.5% and 73.9% in the PEG and non-PEG groups, respectively) (Table 2).

**Table 2:** Treatment during CCRT and outcomes. CCRT: concurrent chemoradiation; CR: complete response; PR: partial response; DP: disease progression

Parameters		Non-PEG group (n=46)	PEG group (n=44)	All (n=90)
Hospitalization during CCRT, n (%)	No	37 (80.4)	39 (90.7)	76 (85.4)
	Yes	9 (19.6)	4 (9.3)	13 (14.6)
Cycles of chemotherapy, n (%)	1	2 (4.3)	0 (0.0)	2 (4.3)
	2	10 (21.7)	13 (29.5)	10 (21.7)
	3	34 (73.9)	31 (70.5)	34 (73.9)
	BMI after CCRT, kg/m^2^ Mean (SD)	21.98 (5.19)	20.60 (4.62)	21.30 (4.93)
Response, n (%)	CR	31 (75.6)	29 (69.0)	60 (72.3)
	PR	10 (24.4)	12 (28.6)	22 (26.5)
	DP	0 (0.0)	1 (2.4)	1 (1.2)
Disease progression after CCRT, n (%)	No	31 (67.4)	29 (65.9)	60 (66.7)
	Yes	15 (32.6)	15 (34.1)	30 (33.3)
Death, n (%)	No	32 (69.6)	31 (70.5)	63 (70.0)
	Yes	14 (30.4)	13 (29.5)	27 (30.0)

ANCOVA analysis revealed that there was an effect of the covariate baseline BMI on BMI at the end of CCRT (F {1.84}=518.814 {p=0.000}). There wasn't an effect of PEG placement on BMI at the end of CCRT after controlling for the effect of baseline BMI (F {1.84}=0.065 {P=0.799}). BMI was significantly lower after CCRT for the total study population (21.30 kg/m^2^ vs. 23.97 kg/m^2^), t(86)=12.389, p<0.001. In the subgroup with baseline BMI <18.5 kg/m^2^ (15 patients), 90% of patients with prophylactic PEG completed the three planned chemotherapy cycles vs. 66.7% in the non-PEG group.

**Figure 1:**
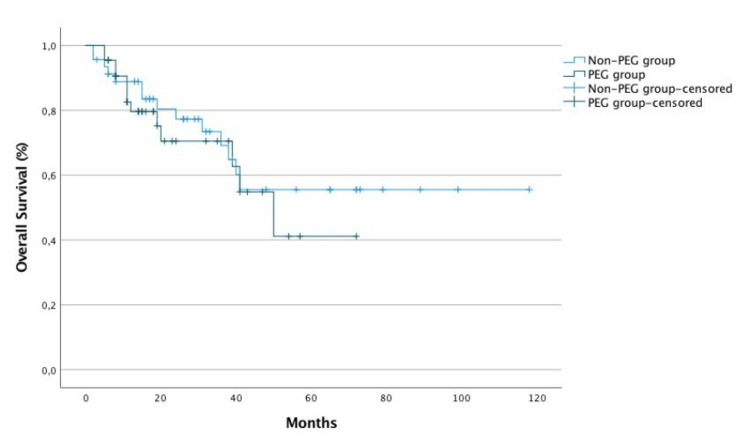
Overall survival according to prophylactic percutaneous endoscopic gastrostomy placement.

**Figure 2:**
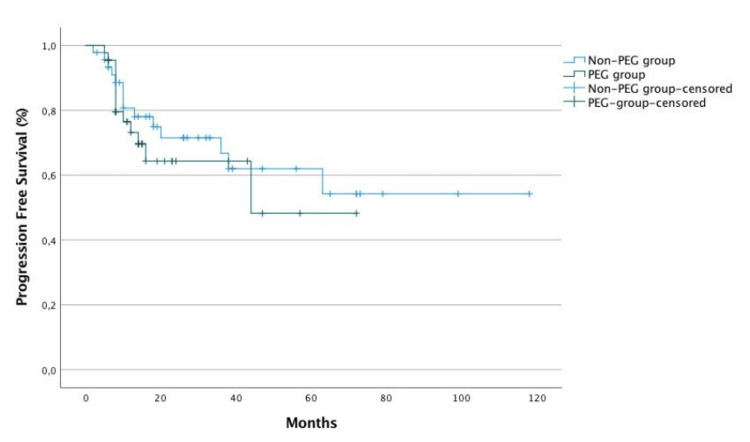
Progression-free survival according to prophylactic percutaneous endoscopic gastrostomy placement.

Ten patients in the PEG group (22.7%) referred feeding tube dependency. Patients with dysphagia were 3.2 times more likely to have placed prophylactic PEG (p=0.007). The difference in overall survival (OS) and progression-free survival (PFS) between the two groups was not statistically significant (p=0.57 and p=0.497, respectively) (Figures 1, 2).

## Discussion

Patients with locally advanced HNC who undergo CCRT often develop malnutrition because oral feeding may be diminished due to pain and other treatment toxicities. Thus, placement of prophylactic PEG before starting treatment can be equated to guarantee adequate supply, avoid weight loss, and increase treatment tolerance. Nonetheless, few real-world data studies have investigated the utility and benefits of prophylactic PEG [18].

In this study, we aimed to evaluate the use of prophylactic PEG in a cohort of patients with locally advanced HNC who underwent CCRT. In the PEG group, 77.3% of patients were not underweight, so the placement of PEG was to avoid malnutrition, which we consider concordant with the aim of the study. Most patients in our study were diagnosed with stage III-IVa HNC. Although we found that significantly more patients in the PEG group had symptoms of dysphagia, there were no significant differences in the other baseline clinical characteristics, including age, baseline BMI, nodal, and TNM stage between the two groups, which was not concordant with the findings in a preceding study which concluded that higher nodal stage and older age were independent predictors associated with a need for placement of prophylactic PEG [18].

In the study population, BMI was significantly lower after CCRT, which was expected according to the literature [18]. However, there was no significant effect of PEG tube placement on BMI at the end of CCRT, after controlling for the baseline BMI effect. Regarding survival outcomes, there was no significant difference in OS and PFS between the two groups. On one hand, this may be because the sample is small, and more patients are needed for the difference to gain significance. On the other hand, most patients with PEG had a good nutritional status and performed the procedure with a prophylactic purpose. This may mean that the majority managed to maintain a good oral nutritional intake, at par with the group that did not have PEG. In addition, most patients were evaluated in a nutrition appointment, which is an important factor in terms of ensuring that even if the oral intake is smaller, it is carried out with the right diet that is more nutritious and protein-rich in order to improve nutritional status. We call attention to the importance of early referral and evaluation in this consultation of all patients with advanced disease. Patients with dysphagia were 3.2 times more likely to have placed prophylactic PEG. This alerts us to the importance of the presence of dysphagia at the time of diagnosis.

Ten patients in the PEG group (22.7%) referred feeding tube dependency. This is a possible consequence described that we should investigate in the follow-up consultations so that an early referral to speech therapy and physiotherapy can be carried out in order to improve chewing and swallowing with the necessary functional intervention. Hospitalization during CCRT was not significantly higher in the non-PEG group, as reported by Bojaxhiu et al. [23]. This study concludes that omitting prophylactic PEG tube placement is an option in patients being carefully monitored without increasing the risk of unplanned hospitalization due to dysphagia, dehydration, or malnutrition [23].

In the subgroup with baseline BMI <18.5 kg/m^2^, 90% of patients with prophylactic PEG were able to complete the three planned cycles of chemotherapy vs. 66.7% that had not been subjected to prophylactic PEG. According to the literature, performing more than 200 mg/m^2^ of chemotherapy with cisplatin during CCRT has an impact on overall survival [24]. Therefore, despite the need for a larger number of patients in this subgroup for us to have statistically significant conclusions, we consider that these data may be relevant. This subgroup of patients may be the one in which the use of prophylactic PEG may be more beneficial in terms of treatment compliance and, consequently, survival outcomes.

However, we recognize some limitations in this study. This is a multicenter study, so an imbalance in baseline characteristics and treatment received per institutional guidelines unavoidably occurred. In addition, we were faced with selection bias of the treating physicians and some missing data, as this is a retrospective study. Finally, we could not collect data on PEG-related complications, which could also be useful in this discussion.

## Conclusions

Patients with locally advanced HNC performing CCRT had a significant impact on weight and, consequently, BMI. Patients with dysphagia were more likely to have placed prophylactic PEG before CCRT. In this study using real‐world data, we found a potentially protective effect of PEG in underweight patients in order to complete three cycles of treatment. These results can help guide clinical practice by promoting new information and assisting the discussion of cases and decisions in multidisciplinary meetings. Prospective studies with larger samples are needed to validate these conclusions.
